# Diathermy

**Published:** 1913-05-17

**Authors:** Alfred C. Norman

**Affiliations:** House Surgeon at Durham County Eye Infirmary.


### ELECTRICITY IN MODERN MEDICINE.*

### XXIX.—Diathermy.

By ALFRED C. NORMAN, M.D. Edin., House Surgeon at Durham County Eye Infirmary.

The currents used in diathermy are high-frequency currents generated by a special apparatus furnishing a large amperage, a low voltage, and practically undamped oscillations. Ordinary high-frequency currents, generated by a D'Arsonval transformer, have a voltage of about 100,000, and a maximum available amperage of only about .5 (500 milliamperes), whereas diathermy currents have a voltage not exceeding 2,000, and an available current of fully three amperes. The oscillations follow each other at a rate of perhaps three millions per second, and the waves are perfectly regular, consequently currents of great magnitude (three amperes or more) can be applied to the human body without giving rise to the phenomena ordinarily associated with electricity. There is no pain, no muscular contraction, and no electrolysis; all we notice is a rise of temperature directly proportional to the amperage traversing the tissues.

### The Diathermy Outfit.

A technical description of the apparatus used in diathermy would occupy more space than I have at my disposal in these articles; it is less complicated and considerably more portable than the ordinary high-frequency apparatus; in fact, it somewhat resembles in appearance the Pantostat mounted on a trolley. In places where the main supply is alternating the diathermy apparatus may be connected directly to the electric lighting wires, but where a continuous current only is available a motor transformer must be used to convert the continuous current into an alternating one. On the instrument there are cranks for varying the current strength, terminals for connecting with the patient, and an ampere-meter for measuring the quantity of current the patient receives. The apparatus has, perhaps, one disadvantage (which is, of course, infinitesimal compared with its advantages), and that is that in treating certain neurotic cases where suggestion is of value, the simple exterior of the diathermy outfit is not as imposing as that of the old high-frequency apparatus.

In diathermy we have a means of raising any portion of the human body to almost any desired temperature. The heat is not produced at the expense of the individual as in the case of inflammation; it does not enter the body by conduction, as in the case of poultices and the like, which affect only the superficial tissues, but it represents fresh energy brought into the body by the electric current, and having a potent action for good or evil upon every single cell that is traversed by the current.

The thermal action of these currents promises to be of very great value in both medicine and surgery. In the treatment of inflammatory conditions the action can be accurately localised to the part involved; vaso-dilatation is produced, increased absorption follows, the growth of micro-organisms is inhibited, and relief from pain is the almost invariable rule. In surgery the currents can be so intensified as to produce actual destruction of tissue.

### Therapeutic Applications of Diathermy.

In giving therapeutic applications the following points must be borne in mind: (1) The current passes straight through the tissues from electrode to electrode (*i.e.*, it is not diffused through a large area of the body as in the case of ordinary electricity), consequently the area acted upon depends upon the size of the electrodes. (2) The intensity of action depends upon the *strength* of current used, upon the *time* it is flowing, and upon the *area* of tissue through which it passes—in other words, upon the size of the electrodes. For instance, one ampere applied through an electrode one inch square would produce in a given time far more reaction in the tissues beneath it than it would if applied through an electrode four inches square.

Remembering these points, we are in a position to obtain the exact results we require simply by selecting electrodes of suitable size and by varying the strength of the current. For instance, to apply diathermy to an inflamed elbow we should select two electrodes, made of flexible metal and covered with lint well moistened in salt solution, of about the same size as the area to be treated. We should apply these to opposite sides of the joint, care being taken that they are in close apposition to the skin in order to obtain uniform current density, connect them by flexible wires to the terminals of the apparatus and allow a current of from .5 to 1 ampere to flow until sufficient heat is produced. There is no organ of the body that cannot be treated similarly, but in the case of large organs—the liver, for instance—larger electrodes must be used, and a stronger current will be required to produce the same amount of heat.

Nagelschmidt of Berlin claims to have obtained marvellous results by means of diathermy. All painful affections of the nerves and joints are relieved; sciatica, lumbago, neuralgia, tic douloureux, tabetic pains and gout all respond in a remarkable manner to this method of treatment. He suggests its use in pleurisy, pneumonia, cholecystitis, and in various affections of the eye and ear, but advises caution in the treatment of phthisis because of the danger of hæmorrhage following the hyperæmia. It is in circulatory disorders that Nagelschmidt has obtained his happiest results. Good results have been obtained in the treatment of venous stasis, varicose veins and hæmorrhoids. High arterial tension has been reduced in a remarkable manner.

* Previous articles appeared on Nov. 11, 25, Dec. 9, 30, 1911; Jan. 13, 27, Feb. 17, March 9, 30, April 20, May 4, 25, June 8, July 6, Aug. 3, 17, 31, Sept. 28, Oct. 12, 26, Nov. 9, 30, Dec. 14, 1912; Jan. 11, Feb. 1, March 1, April 12, and May 3.

Cases of aortic aneurism, attacks of heart failure, arterio-sclerosis, and intermittent claudication have all derived marked benefit from diathermy, while the results obtained in cases of angina pectoris are little short of miraculous.

Surgical Applications of Diathermy.

When it is desired to destroy tissue, as in the case of malignant growths, lupus, and the like, it is only necessary to increase the strength of the current and to reduce the size of the active electrode until the current is sufficiently concentrated to produce actual heat-coagulation in the tissues under the active electrode. The neutral electrode is placed on the opposite side of the part and, being much larger, has no destructive action.

Nagelschmidt claims the following advantages for diathermy in surgery: (1) Extensive operations may be done with a minimal loss of blood owing to the fact that all vessels are sealed by heat coagulation. (2) Infected masses are rendered sterile before removal. (3) In operating on cancer the spread of cancerous cells into blood vessels or lymph channels is prevented by the sealing of the latter. (4) Some parts inaccessible to the knife can be reached by diathermy—*e.g.*, cancer of the bones of the face. (5) The hyperæmia that follows a diathermic operation flushes out all toxic products and rapidly promotes healing.

Diathermy should not be confounded with fulguration. Fulguration consisted in destroying tissue by means of powerful sparks from an Oudin resonator, and has now been practically discarded. Diathermy is heat coagulation produced in the tissues as a result of a very concentrated current, not by sparking. The usual method is to coagulate to a depth of 1 cm. and then remove the layer with a blunt curette or a swab, proceeding layer by layer until the whole growth is destroyed. In deep-seated tumours the growth is exposed by the knife before diathermy is attempted. Its applications in surgery are too numerous to consider in detail, but it promises to be particularly useful in gynæcology, diseases of the nose and throat, cancer, lupus, tuberculosis of mucous membranes, hæmorrhoids, and nævi.

The present writer is indebted to the writings of Nagelschmidt and Howard Humphris for much information on this fascinating subject, and those who are interested should refer to articles by them in the *Archives of the Röntgen Ray.**

* June 1910, July 1910, September 1910, December 1910, February 1912, July 1912.

